# Automated Capture of Intraoperative Adverse Events Using Artificial Intelligence: A Systematic Review and Meta-Analysis

**DOI:** 10.3390/jcm12041687

**Published:** 2023-02-20

**Authors:** Michael B. Eppler, Aref S. Sayegh, Marissa Maas, Abhishek Venkat, Sij Hemal, Mihir M. Desai, Andrew J. Hung, Teodor Grantcharov, Giovanni E. Cacciamani, Mitchell G. Goldenberg

**Affiliations:** 1 Catherine and Joseph Aresty Department of Urology, Keck School of Medicine, University of Southern California, Los Angeles, CA 90033, USA; 2 Department of Surgery, Clinical Excellence Research Centre, Stanford University, Stanford, CA 94305, USA

**Keywords:** artificial intelligence, intraoperative adverse events, systematic review, PRISMA, adverse effects, intraoperative period

## Abstract

Intraoperative adverse events (iAEs) impact the outcomes of surgery, and yet are not routinely collected, graded, and reported. Advancements in artificial intelligence (AI) have the potential to power real-time, automatic detection of these events and disrupt the landscape of surgical safety through the prediction and mitigation of iAEs. We sought to understand the current implementation of AI in this space. A literature review was performed to PRISMA-DTA standards. Included articles were from all surgical specialties and reported the automatic identification of iAEs in real-time. Details on surgical specialty, adverse events, technology used for detecting iAEs, AI algorithm/validation, and reference standards/conventional parameters were extracted. A meta-analysis of algorithms with available data was conducted using a hierarchical summary receiver operating characteristic curve (ROC). The QUADAS-2 tool was used to assess the article risk of bias and clinical applicability. A total of 2982 studies were identified by searching PubMed, Scopus, Web of Science, and IEEE Xplore, with 13 articles included for data extraction. The AI algorithms detected bleeding (n = 7), vessel injury (n = 1), perfusion deficiencies (n = 1), thermal damage (n = 1), and EMG abnormalities (n = 1), among other iAEs. Nine of the thirteen articles described at least one validation method for the detection system; five explained using cross-validation and seven divided the dataset into training and validation cohorts. Meta-analysis showed the algorithms were both sensitive and specific across included iAEs (detection OR 14.74, CI 4.7–46.2). There was heterogeneity in reported outcome statistics and article bias risk. There is a need for standardization of iAE definitions, detection, and reporting to enhance surgical care for all patients. The heterogeneous applications of AI in the literature highlights the pluripotent nature of this technology. Applications of these algorithms across a breadth of urologic procedures should be investigated to assess the generalizability of these data.

## 1. Introduction

Intraoperative complications account for 48% of all preventable adverse events in hospitalized patients [1] and may have a significant clinical and fiscal impact on the post-operative course [2,3,4]. Despite their ubiquity, intraoperative adverse events (iAEs) are inadequately collected in practice and serially underreported in the literature [5,6], limiting our understanding of their role in determining surgical outcomes and limiting our efforts at mitigating their occurrence. 

The paucity of data surrounding iAEs stems from several limitations in the way that we routinely detect and capture these events. Traditional efforts to study iAEs have relied on retrospective review of medical records, incident/operative reports, and patient safety databases, often in the setting of a significant post-operative complication (i.e., death or re-operation) [2,7]. This approach is often limited by recall bias, selection bias, and by incomplete records [2]. Retrospective iAE detection makes it challenging to capture and evaluate clinically significant events that may have caused patient harm without leading to a pre-defined outcome of interest (i.e., “near-miss events”) [2]. Prospective, real-time observation and capture of iAEs is a more sensitive and accurate method for capturing the full range and impact of iAEs [7] but is limited by logistical, technological, and cost constraints. Furthermore, the lack of standardized definitions and guidelines for reporting iAEs hinders the collection and study of iAEs, even when performed in a prospective fashion. These limitations contribute to the underreporting of iAEs and highlight a need for better tools for the detection and study of iAEs, ideally in a proactive, structured, and standardized fashion.

There may be an emerging role for artificial intelligence (AI) in this role. Recent deep learning and computer vision algorithms, for instance, have already shown promise in helping identify dangerous anatomic planes [8] and potential surgical missteps [9] in the real-time analysis of laparoscopic surgical video. These efforts highlight a potential utilization of AI within the operating room in proactively detecting and recording iAEs.

Therefore, the purpose of this systematic review is to evaluate the medical and biomedical engineering literature to examine the clinical use of automated methodologies for the detection, collection, and analysis of iAEs broadly across all surgical fields and to assess the quality of the current state of research.

## 2. Materials and Methods

A systematic review of the published literature on the automatic detection of iAEs using AI was conducted according to the Preferred Reporting Items for Systematic Reviews and Meta-Analysis (PRISMA) statement [10] and the PRISMA diagnostic test accuracy (DTA) statement [11,12] (File S1). The systematic review was registered and approved through PROSPERO [13] (ID# CRD42022353402).

### 2.1. Search Strategy

A comprehensive search was performed on 10 August 2022, in the databases PubMed, Scopus, Web of Science, and IEEE Xplore. A combination of MeSH and free text terms were used. The following terms were used in combination and synonyms were also included in the search: “Artificial Intelligence”, “surgery”, “adverse event”, and “predict”. Publication date was restricted from 2010 to current. The complete search terms are shown in Table S1.

### 2.2. Inclusion and Exclusion Criteria

Included articles (1) were from any surgical specialty, (2) included the intraoperative phase of care only, (3) described any iAE as defined by the Harvard Medical Practice Study as “an injury that was caused by medical management (rather than the underlying disease) and that prolonged the hospitalization, produced a disability at the time of discharge, or both”, (4) identified iAEs using an automated or real-time method (i.e., AI-based), (5) were in English, and (6) were from any published abstract or full manuscripts, including prospective and retrospective case series, cross-sectional studies, clinical trials, and systematic reviews or meta-analyses. We included studies even if they were not in human models, to provide as many examples of potential AI applications in automatic intraoperative adverse event detection as possible.

Articles were excluded if they (1) had no full text, (2) included pre- or postoperative phases of care, (3) were from nonsurgical specialties, (4) used non-automated data collection methods, (i.e., human rater-based review of surgical video), or (5) were papers without original data, including editorials, letters to the editor, and comments.

### 2.3. Screening

Covidence software was utilized for the title/abstract and full-text screen. After importing articles and the removal of duplicates, two reviewers (ME/AS) independently screened titles and abstracts based on inclusion/exclusion criteria. In situations where a paper was considered potentially relevant based on its title and abstract, it was included for full-text review. A third reviewer (MG) acted as a mediator in cases of disagreement. Full-text screening was also independently conducted by two independent reviewers (ME/AS), again mediated by a third reviewer (MG). 

### 2.4. Data Extraction

Two independent reviewers (ME/AS) utilized a standardized excel document to extract relevant data from the included articles. Data extracted included information on study methodology, study sample size, type of adverse event under study, type of AI used, and information on application of the AI for detection of the adverse event [14]. Following this, a third reviewer (MG) reviewed the data and corresponding articles for any inconsistencies.

### 2.5. Meta-Analysis

Algorithms from included studies with available data (prevalence, sensitivity, and PPV) were included in the meta-analysis [15]. Forest plots were generated to assess detection heterogeneity and variability in sensitivity and specificity of these algorithms. A hierarchical summary ROC was created to evaluate the overall ability of these algorithms to predict the iAE of interest in the respective study. 

### 2.6. Quality Assessment

The study used the QUADAS-2 tool to assess the quality of the included diagnostic accuracy studies [16]. We followed the four phases as recommended: (1) state the review question, (2) develop review specific guidance, (3) review published or create unique flow diagram, (4) judge the bias and applicability of each study. We used signaling questions provided by QUADAS when rating study bias risk as high, low, or unclear. For each key domain (patient selection, index test, reference standard, and flow of patients through study), the study was graded as low risk for bias if corresponding signaling questions were answered with “yes”, the study was graded as high risk for bias if at least one corresponding signaling question was answered with “no”, and the study was graded as unclear risk of bias if at least one signaling question was answered with “unclear”. The QUADAS-2 tool recommends creating flow-charts or using flow-charts if published by articles to aid in determining study bias and predictability.

## 3. Results

**Figure 1:**
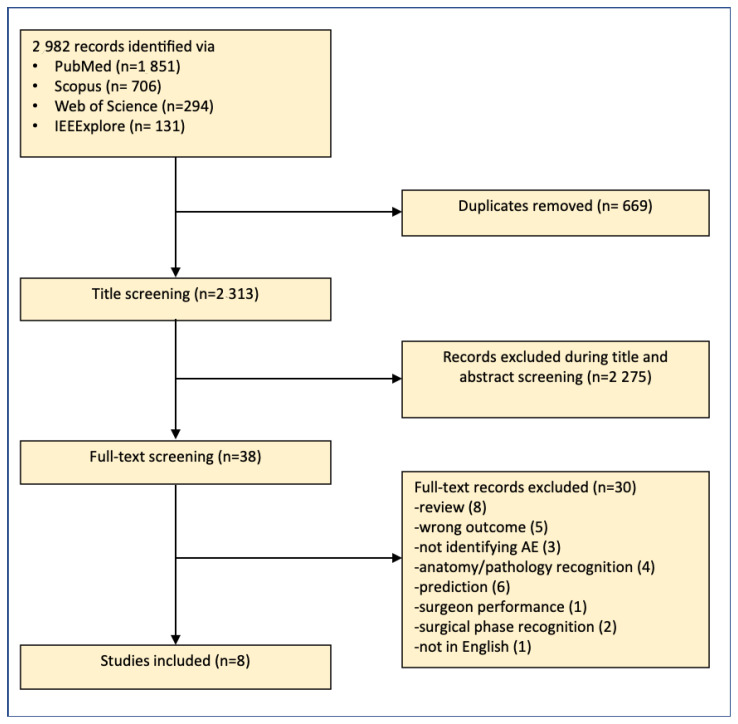
Flow diagram: study identification and screening process.

**Figure 2:**
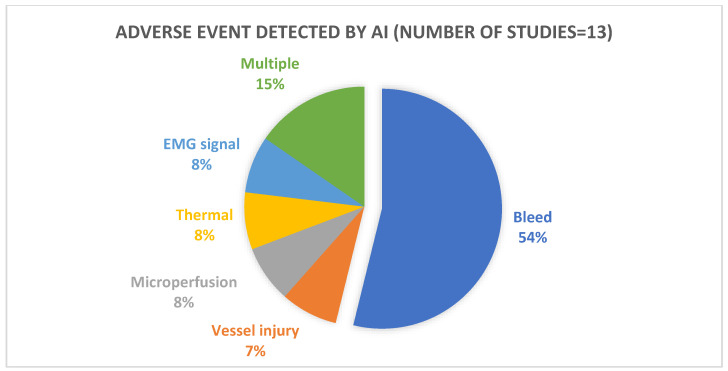
Intraoperative adverse event type identified by artificial intelligence (AI).

A total of 2982 studies were identified through searching PubMed, Scopus, Web of Science, and IEEE Xplore. After the removal of 669 duplicates, 2313 studied were included for title and abstract screening. Of those, 2275 studies were identified as irrelevant and excluded, while 38 were included for full-text assessment for eligibility. Of those, eight articles were included for data analysis. Furthermore, review of the included articles and subsequent literature searches identified five articles that were independently assessed by reviewers (ME/AS) and were determined to be eligible for inclusion. A flow-chart of the article selection process is shown in Figure 1. The 13 studies included in this review were published from 2016 to 2022 [17,18,19,20,21,22,23,24,25,26,27,28,29], with the majority published during or after 2020. Table 1 shows an overview of the methodologies used, adverse events analyzed, AI algorithms, type of validation, outcomes, and comparative metrics from the 13 included articles. Table 2 summarizes the various types of algorithms used in the development of the automatic iAE identification systems. The AI systems in the included articles detect bleeding (seven articles), vascular/vessel injury (one article), perfusion deficiencies (one article), thermal damage (one article), EMG abnormalities (one article), and multiple iAEs (two articles) (Figure 2). 

### 3.1. Outcome Statistics

All 13 articles reported outcome statistics for the automatic detection of iAEs in terms of sensitivity, specificity, accuracy, and/or AUR-ROC. Twelve of the thirteen articles reported sensitivity, specificity, and/or accuracy. More studies (nine) reported sensitivity than specificity (five). Not including sensitivity values of unoptimized versions of algorithms and sensitivity values based on single patient data, the range of sensitivities was 0.7–1.0. A summary of the outcome statistics from each study is shown in Table S2.

Five of the thirteen studies calculated an AUC-ROC value. The reported AUC-ROC was lowest (0.82) for bleeding detection in Wei et al. [24] and highest (0.97) in Morita et al.’s study of iAE detection in cataract surgery [18]. 

### 3.2. Study Validation and Conventional Parameters

There was significant heterogeneity in the validation methodology across the included articles. Nine of the thirteen articles described validating the detection system, while five of the thirteen articles reported using cross-validation for the detection system. In at least two articles, cross-validation was used to establish a threshold for determining a positive or negative outcome. Seven studies divided data into training and validation cohorts, thereby providing external validation of their data [30]. 

Six of the thirteen studies compared the AI outcome statistics to a conventional parameter, previously used metric, or other control (Table 1). Examples include time ratio (TR) and rising slope (RS) for organ microperfusion, B-Mode Ultrasound (US) for thermal lesions, and controls for predicting blood loss.

### 3.3. Meta-Analysis

**Figure 3:**
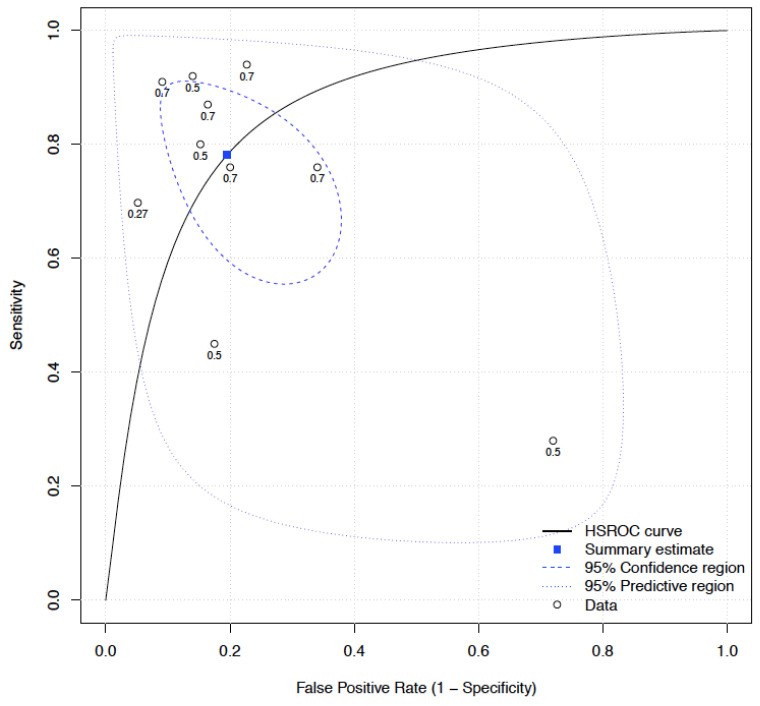
Random effects meta-analysis of intraoperative adverse events machine learning algorithms. HSROC: hierarchical summary receiver operating characteristics.

A meta-analysis was conducted, combining model performance data from a total of ten algorithms in four of the included articles. The grouped sensitivity was 0.78 (CI 0.64–0.88) and specificity was 0.81 (CI 0.69–0.88) (Figures S1 and S2) of these algorithms. A hierarchical summary ROC was created to calculate the combined ability to predict iAEs, which included ophthalmological, vascular, bleeding, and intraoperative EMG abnormalities. Overall, these algorithms were highly predictive for the iAEs (OR 14.74, CI 4.70–46.18) (Figure 3). 

### 3.4. Quality Assessment

**Figure 4:**
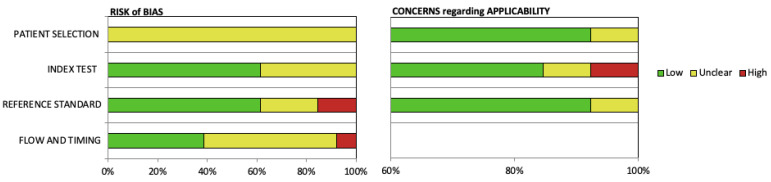
QUADAS-2 grading of article risk of bias and concerns regarding applicability (n = 13).

**Figure 5:**
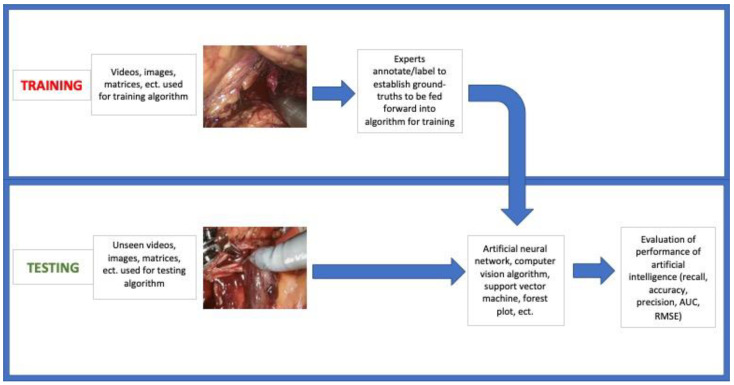
Flow-chart depicting methods of included articles, generally applicable to all included studies. AUC area under curve, RMSE root–mean square error.

A summary of the risk of bias and applicability based on the QUADAS-2 tool for assessing quality of diagnostic accuracy studies is shown in Figure 4. All studies were rated as unclear risk of bias for patient selection and the majority were rated as unclear for flow and timing. Most studies had a low risk of bias for the index test and reference standard. We rated nearly every study to have low concerns regarding applicability for patient selection, index test, and reference standard. The flow diagram was developed and used to summarize methods of each included article (Figure 5), and the flow-chart created for each article are included in Figure S3.

## 4. Discussion

The surgical community currently lacks a gold standard for objective iAE identification and reporting in the literature, and without standardization, our ability to accurately define surgical complications remains limited. AI represents a potential solution for identifying and capturing iAEs, but has been used sparingly, as demonstrated in this review. The majority of the literature in this review was published after 2020, highlighting the novelty of this work relative to other AI applications in surgical science. Different forms of deep neural learning algorithms were employed in the surveilled papers, including SVM, Inception V3, and CNN, as well as original, novel algorithms. Although bleeding detection was the most common outcome in these studies, several papers demonstrated success in identifying perfusion deficiencies, thermal damage, and EMG abnormalities. Included surgical specialties were urology, ophthalmology, general surgery, and neurosurgery. This heterogeneity of iAEs and subspecialties included in this review speaks to the generalizability of many of these methodologies, and lays a promising foundation for the future use of AI in iAE reporting. 

For AI to successfully identify iAEs over the course of a procedure, the algorithm must first be able to recognize what is expected and routine. There are several instances in the literature where AI has demonstrated success in analyzing and correctly identifying both surgical steps and outcomes. For example, two distinct neural network models were able to accurately identify laparoscopic instruments and their position in the surgical field using surgical video, calculating measures of surgical efficiency [31,32]. A deep learning algorithm was similarly applied to robotic surgery and able to track the movement of instruments with 83% accuracy [33]. Building on this work, Hashimoto and colleagues used a neural network trained with computer vision data to autonomously annotate the procedural steps of a general laparoscopic procedure with accuracy in the mid-1980s [9]. Taken together, the ability for AI models to identify surgical stages, surgical instruments, and instrument usage lays the framework for the AI-based identification of deviations from standard procedure, including suboptimal instrument usage and aberrations in the flow of surgical steps. 

AI has been used successfully to measure surgical efficiency and improve skills [34,35,36,37,37,38]. Specifically, CNNs use feature extraction to provide objective feedback for trainees on robotic skills such as suturing, knot tying, and needle passing with accuracy in the 90s [39], and entropy-based models achieves similar levels of success in the automated scoring of suturing and knot-tying [40]. Computer vision has been similarly employed to critique open surgical skills based on ratings from expert surgeons [41]. Kinematic and surgical error data take robotic surgery feedback one step further with the addition of objective kinematic data to subjective, expert-derived scoring of intraoperative video [42]. In fact, tool motion tracking, hand motion tracking, eye motion tracking, and muscle contraction data can all be incorporated into machine and deep learning models to predict a surgeon’s skill level [43]. 

While intraoperative events account for a proportion of the variability in patient safety and surgical outcomes, clinicopathological factors certainly play a significant role as well. Preoperative patient data have traditionally been used in predictive models evaluating postoperative outcomes in urologic and other surgical procedures [44,45]. While these methods for evaluating perioperative risk for patients will continue to be used and enhanced with AI, only when combining preoperative and intraoperative data will our ability to accurately anticipate postoperative complications be optimized. 

The underreporting of iAEs is a multifactorial issue. Most notably, there is a lack of a standardized, accurate, and reproducible definition in the surgical literature [46]. Studies suggest that the medical record is an unreliable source of iAE data, even with regards to ‘never events’ [47,48]. Furthermore, data suggest that not all surgeons disclose iAEs to patients, and this lack of communication further obscures iAE reporting in the literature, possibly as a result of the negative impact these events have on a surgeon’s psyche [49]. AI may help bridge this gap by identifying iAEs directly from operative footage as opposed to relying on retrospective collection such as EMR abstraction. A byproduct of improved reporting is potential improvements in the counselling of patients preoperatively, and more realistic rates of iAEs may encourage surgeons to be more forthcoming in disclosing these events. The most promising use of these data is the development of strategies that allow for the mitigation or minimization of iAEs in regard to safety and surgical outcomes. Only with the comprehensive capture of any event that threatens patient safety, both resulting in complications and ‘near misses’, can we uncover the most suitable educational and quality improvement interventions to enhance our care of surgical patients.

Our current, primary approach to discussing iAEs in clinical practice are morbidity and mortality conferences (MMCs). These remain a cornerstone in departmental and hospital quality improvement for analyzing patient cases with suboptimal outcomes, normally identified as a result of an unusual or concerning complication in the postoperative period. However, accurate surgical recall fades with time, limiting the ability of MMCs to bring about constructive changes to patient care [50]. Additionally, a minority of complications occurring in the process of clinical practice are discussed at MMCs, introducing selection bias into this process [51,52]. Despite these challenges, MMCs are perceived as successful and important to medical education, and efforts to improve them should be undertaken [30]. Prospective, robust methods of identifying iAEs would greatly enhance these initiatives, including the use of video feedback and AI feature analysis of these events helping minimize inherent errors of human recall. 

Limitations of this systematic review include the heterogeneity of the AI models employed and the robustness of the model validation strategies used. Additionally, many models used in the review were developed with data from small numbers of individual expert opinions, potentially limiting the generalizability of those models across different patient populations. Variability in the types of iAEs included in each study serves as both a strength and limitation, and further work is needed to establish and validate appropriate models that can accurately capture the wide range of iAEs encountered during surgical procedures. While a meta-analysis was able to demonstrate the overall combined performance of the published AI algorithms, the variations in the outcomes of interest and internal/external validation techniques used need to be accounted for when interpreting these findings. Additionally, the data used in the meta-analysis were extracted from the articles themselves, without access to the raw data. Finally, this review is limited by the overall quality of the studies and there is a need for randomized studies.

## 5. Conclusions

There is a demonstrable need for the standardization of iAE identification and reporting in surgery, which may be improved with the incorporation of AI technology. While the models included in this review provide a promising foundation for the use of AI software in iAE reporting, rigorous testing of these models in larger, diverse populations is paramount. For universal iAEs, such as blood loss, existing models should be tested across different surgical specialties. Additionally, established models should be tested on different procedures within the same specialty to identify models that are more broadly applicable.

## Figures and Tables

**Table 1:** Summary of the 13 included articles.

Author (Year)	Study Type, Data Source	Dataset	Type of Procedure, Surgical Specialty	Adverse Event, Medium	Type of AI	AI Training/Ground-Truth Establishment	Validation	Outcome/Comparison to Ground-Truth or Conventional Parameter
Chen et al. [17] (2021)	Retrospective Data source: Recorded videos of 50 different TURP procedures	287 video clips from complete recording videos of 50 different TURP procedures 150 videos training data (10% for validation); 137 videos testing data	TURP, urology	Bleeding, video	ResUNet for segmentation (neural network) KNN, NB, Random Forest, SVM for video classification	3 experienced urologists graded video clips 0–3 based on visual clarity	Validation completed (limited information), unique data for testing stage	KNN: highest performing AI classification model; Improved when compared to ground-truth after optimizing video
Morita et al. [18] (2020)	Retrospective Data source: Recordings of cataract surgeries performed at Saneikai Tsukazaki Hospital	425 video recordings of cataract surgery 310 training data (57 with problems), 15 validation data (5 problems), 100 test data (50 with problems)	Cataract surgery, ophthalmology	Vitreous prolapse, capsule rupture, damage to iris, iris prolapse, rupture of the zonule of the zinn, dropped nucleus, video	Inception V3 (neural network)	Annotations of surgical problems in video of cataract surgery	Validation completed (limited information), unique data for testing stage	High problem detection in critical phase of cataract surgery; detected problem faster than ophthalmologist 42/44 (95%) times
Park et al. [19] (2020)	Prospective Data source: Patients undergoing laparoscopic surgery for colorectal cancer at Pusan National University Yangsan Hospital	50 training videos (10,000 ICG curves from 200 different locations in the ICG videos) 15 testing videos	Laparoscopic surgery for colorectal cancer, general surgery	Microperfusion, Indocyanine green (ICG) curves	Self-organizing map (neural network)	Training ICG curves were classified into 25 most common patterns, associated with risk of inadequate perfusion	Cross-validation, unique data for testing stage	Compared to T 1/2max, TR, and RS, AUC higher (0.842 vs. 0.734, 0.750, 0.677) and equal or higher for most other statistics
Su et al. [20] (2022)	Retrospective Data source: 3 large digital subtraction angiography (DSA) image series databases	4429 patients from 3 databases; 85 perforations, 233 non-perforations in study	Endovascular therapy, interventionalist	Intracranial vessel perforation, DSA runs	Spatial-temporal networks (CNN, RNN)	Experienced radiologist reviewed DSA images for all perforation cases and annotated locations	Ten-fold cross-validation	AI performed at similar level as expert radiologist
Zhang et al. [21] (2019)	Retrospective Data source: 82 groups of ablation experiments from 32 ex vivo liver tissues	1640 ultrasound data matrices of thermal lesions: 1400 for training, 240 for testing	Microwave ablation, n/s	Thermal injuries, ultrasound images	CNN	Optical images of tissues sections used as ground-truths	Validated (limited information)	AUC for AI higher than conventional B-mode images
Zha et al. [22] (2020)	Prospective Data source: EMG data recorded continuously during thyroid surgery	5 patients undergoing thyroid surgery One patient model (85% for training, 15% for testing) Cross-testing (4 for training, 1 for testing)	Thyroid surgery, n/s	Abnormal EMG signals, intraoperative neurophysiological monitoring	CNN, LSTM	Expert neurophysiologists classified EMGs	Unclear validation, unique data for testing stage	AI performed higher than other baseline methods
Garcia-Martinez et al. [23] (2017)	Retrospective Data source: Non-specific laparoscopic videos; in vitro laboratory system	23 in vivo laparoscopic training videos (17 bleeding) In vitro training videos with 5 different configurations 25 in vitro images for testing 32 in vivo images for testing	Various (cholecystectomies, pelvic surgeries, total mesorectal excisions, radical hysterectomies, pancreatectomy, gastrectomy, aortic lymphadenectomy, retroperitoneal dissections, nephroureterectomies, and colectomies)	Bleeding, video	Computer vision algorithm (open source computer vision and machine learning software library Open CV)	Developed algorithm after analyzing series of images for blood detection based on pixel ratios	Cross-validation of pixels to obtain threshold for bleeding, unique date for testing stage	Compared to two previous algorithms for blood pixel classification; in vitro bleeding classification performed better than in vivo bleeding classification
Wei et al. [24] (2021)	Retrospective Data Source: Operating room at St. Michael’s Hospital in Toronto, Canada, using the OR Black Box ^®^	130 laparoscopic videos	Laparoscopic surgery for colorectal cancer, general surgery	Bleeding/thermal injury, video	CNN	Videos reviewed and annotated by three trained surgeons, labeling blood, bleeding, burn, and thermal injury	5-fold cross-validation to select best epoch and threshold, unclear if used unique data for testing	AI outperformed InceptionV3: AUROC 0.74 vs. 0.80 in bleeding detection; 0.83 vs. 0.93 in thermal injury detection; average precision 0.24 vs. 0.36 in bleeding; 0.38 vs. 0.56 in thermal injury detection
Hua et al. [25] (2022)	Retrospective Data source: Laparoscopic surgery video recorded at Peking Union Medical College Hospital	12 bleeding video clips (10 laparoscopic surgeries)	Laparoscopic surgery, general surgery	Bleeding point detection, video	RCNN	Ground-truth areas of bleeding point marked by 2 senior surgeons	No validation explanation	Introduced temporal component that improved bleeding detection compared to previous systems
Okamoto et al. [26] (2019)	Retrospective Data source: Non-specific laparoscopic surgical videos	10 videos of patients undergoing laparoscopic surgery	Laparoscopic surgery, n/s	Bleeding, video	SVM	Ground-truth established by annotations	Cross-validation, unique data for testing stage	High outcome measures when compared to ground-truths
Jo et al. [27] (2016)	Retrospective Data source: Non- specific laparoscopic surgical videos	4 testing videos	Robot-assisted laparoscopy, n/s	Bleeding, video	Original algorithm	Established threshold for hemorrhage candidate areas	No validation explanation, likely unique data for testing stage	No comparison identified
Kugener et al. [28] (2022)	Retrospective Data source: SOCAL	123 training videos, 20 testing videos	Internal carotid artery injury repair, neurosurgery	Bleeding, video	Deep neural network, LSTM	Automated and annotated versions	Validation of model	RSME higher compared to two control methods
Pangal et al. [29] (2022)	Retrospective Data source: SOCAL	127 training videos, 20 testing videos	Internal carotid artery injury repair, neurosurgery	Bleeding, video	Deep Neural Network, LSTM	Blood loss measured for ground-truth	Validated SOCALNet predictions	SOCALNet met or surpassed expert prediction performance

n/s: not-specified; DS: digital subtraction angiography; LSTM: long–short term memory; SVM: support vector machine; CNN: convolutional neural network; RNN: recurrent neural network; KNN: K nearest neighbor; NB: Naïve Bayes; AUC: area under curve; AUROC: area under receiver operating characteristics curve.

**Table 2:** Algorithm Used for Adverse Event Detection.

Algorithm Type	Citation Using Algorithm
Trees and boosting (Random Forest)	17
Support vector machine	17, 26
Naïve Bayes	17
K nearest neighbor	17
Artificial neural network	17, 18, 19, 20, 21, 22, 24, 25, 28, 29
Computer vision algorithm	23, 26, 27

## Data Availability

Not applicable.

## Supplementary Materials

The following supporting information can be downloaded at: https://www.mdpi.com/article/10.3390/jcm12041687/s1, File S1: PRISMA-DTA Checklist; Table S1: Search Terms by Database; Table S2: Study Outcomes; Figures S1 and S2: Forest Plots of sensitivity; Figure S3: Flow-charts of Methodologies for Automatically Detecting Adverse Events.

## References

[1] Leape L.L., Brennan T.A., Laird N., Lawthers A.G., Localio A.R., Barnes B.A., Hebert L., Newhouse J.P., Weiler P.C., Hiatt H. (1991). The nature of adverse events in hospitalized patients: Results of the Harvard Medical Practice Study II. N. Engl. J. Med..

[2] Mitchell I., Schuster A., Smith K., Pronovost P., Wu A. (2016). Patient safety incident reporting: A qualitative study of thoughts and perceptions of experts 15 years after “to err is human”. BMJ Qual. Saf..

[3] Bohnen J.D., Mavros M.N., Ramly E.P., Chang Y., Yeh D.D., Lee J., De Moya M., King D.R., Fagenholz P.J., Butler K. (2017). Intraoperative adverse events in abdominal surgery: What happens in the operating room does not stay in the operating room. Ann. Surg..

[4] Ramly E.P., Larentzakis A., Bohnen J.D., Mavros M., Chang Y., Lee J., Yeh D.D., Demoya M., King D.R., Fagenholz P.J. (2015). The financial impact of intraoperative adverse events in abdominal surgery. Surgery.

[5] Han K., Bohnen J.D., Peponis T., Martinez M., Nandan A., Yeh D.D., Lee J., Demoya M., Velmahos G., Kaafarani H.M. (2017). The surgeon as the second victim? Results of the Boston Intraoperative Adverse Events Surgeons’ Attitude (BISA) study. J. Am. Coll. Surg..

[6] Kaafarani H.M., Velmahos G.C. (2015). Intraoperative adverse events: The neglected quality indicator of surgical care?. Surgery.

[7] Jung J.J., Elfassy J., Jüni P., Grantcharov T. (2019). Adverse events in the operating room: Definitions, prevalence, and characteristics. A systematic review. World J. Surg..

[8] Madani A., Namazi B., Altieri M.S., Hashimoto D.A., Rivera A.M., Pucher P.H., Navarrete-Welton A., Sankaranarayanan G., Brunt L.M., Okrainec A. (2022). Artificial intelligence for intraoperative guidance: Using semantic segmentation to identify surgical anatomy during laparoscopic cholecystectomy. Ann. Surg..

[9] Hashimoto D.A., Rosman G., Witkowski E.R., Stafford C., Navarrete-Welton A.J., Rattner D.W., Lillemoe K.D., Rus D.L., Meireles O.R. (2019). Computer vision analysis of intraoperative video: Automated recognition of operative steps in laparoscopic sleeve gastrectomy. Ann. Surg..

[10] Page M.J., McKenzie J.E., Bossuyt P.M., Boutron I., Hoffmann T.C., Mulrow C.D., Shamseer L., Tetzlaff J.M., Akl E.A., Brennan S.E. (2021). The PRISMA 2020 statement: An updated guideline for reporting systematic reviews. Syst. Rev..

[11] McInnes M.D., Moher D., Thombs B.D., McGrath T.A., Bossuyt P.M., Clifford T., Cohen J.F., Deeks J.J., Gatsonis C., Hooft L. (2018). Preferred reporting items for a systematic review and meta-analysis of diagnostic test accuracy studies: The PRISMA-DTA statement. JAMA.

[12] Salameh J.-P., Bossuyt P.M., McGrath T.A., Thombs B.D., Hyde C.J., Macaskill P., Deeks J.J., Leeflang M., Korevaar D.A., Whiting P. (2020). Preferred reporting items for systematic review and meta-analysis of diagnostic test accuracy studies (PRISMA-DTA): Explanation, elaboration, and checklist. BMJ.

[13] Mitchell Goldenberg M.E., Aref S., Giovanni C. (2022). Automated Capture of Intraoperative Adverse Evenets: A systematic Review. PROSPERO Int. Prospect. Regist. Syst. Rev..

[14] Stam W.T., Goedknegt L.K., Ingwersen E.W., Schoonmade L.J., Bruns E.R., Daams F. (2021). The prediction of surgical complications using artificial intelligence in patients undergoing major abdominal surgery: A systematic review. Surgery.

[15] Russo G.I., Sholklapper T.N., Cocci A., Broggi G., Caltabiano R., Smith A.B., Lotan Y., Morgia G., Kamat A.M., Witjes J.A. (2021). Performance of narrow band imaging (Nbi) and photodynamic diagnosis (pdd) fluorescence imaging compared to white light cystoscopy (wlc) in detecting non-muscle invasive bladder cancer: A systematic review and lesion-level diagnostic meta-analysis. Cancers.

[16] Whiting P.F., Rutjes A.W., Westwood M.E., Mallett S., Deeks J.J., Reitsma J.B., Leeflang M.M., Sterne J.A., Bossuyt P.M. (2011). QUADAS-2: A revised tool for the quality assessment of diagnostic accuracy studies. Ann. Intern. Med..

[17] Chen J.-W., Lin W.-J., Lin C.-Y., Hung C.-L., Hou C.-P., Tang C.-Y. (2021). An Automatic Bleeding-Rank System for Transurethral Resection of the Prostate Surgery Videos Using Machine Learning. Diagnostics.

[18] Morita S., Tabuchi H., Masumoto H., Tanabe H., Kamiura N. (2020). Real-time surgical problem detection and instrument tracking in cataract surgery. J. Clin. Med..

[19] Park S.-H., Park H.-M., Baek K.-R., Ahn H.-M., Lee I.Y., Son G.M. (2020). Artificial intelligence based real-time microcirculation analysis system for laparoscopic colorectal surgery. World J. Gastroenterol..

[20] Su R., van der Sluijs M., Cornelissen S.A., Lycklama G., Hofmeijer J., Majoie C.B., van Doormaal P.J., van Es A.C., Ruijters D., Niessen W.J. (2022). Spatio-temporal deep learning for automatic detection of intracranial vessel perforation in digital subtraction angiography during endovascular thrombectomy. Med. Image Anal..

[21] Zhang S., Wu S., Shang S., Qin X., Jia X., Li D., Cui Z., Xu T., Niu G., Bouakaz A. (2019). Detection and monitoring of thermal lesions induced by microwave ablation using ultrasound imaging and convolutional neural networks. IEEE J. Biomed. Health Inform..

[22] Zha X., Wehbe L., Sclabassi R.J., Mace Z., Liang Y.V., Yu A., Leonardo J., Cheng B.C., Hillman T.A., Chen D.A. (2020). A deep learning model for automated classification of intraoperative continuous emg. IEEE Trans. Med. Robot. Bionics.

[23] Garcia-Martinez A., Vicente-Samper J.M., Sabater-Navarro J.M. (2017). Automatic detection of surgical haemorrhage using computer vision. Artif. Intell. Med..

[24] Wei H., Rudzicz F., Fleet D., Grantcharov T., Taati B. (2021). Intraoperative Adverse Event Detection in Laparoscopic Surgery: Stabilized Multi-Stage Temporal Convolutional Network with Focal-Uncertainty Loss. Proc. Mach. Learn. Healthc. Conf..

[25] Hua S., Gao J., Wang Z., Yeerkenbieke P., Li J., Wang J., He G., Jiang J., Lu Y., Yu Q. (2022). Automatic bleeding detection in laparoscopic surgery based on a faster region-based convolutional neural network. Ann. Transl. Med..

[26] Okamoto T., Ohnishi T., Kawahira H., Dergachyava O., Jannin P., Haneishi H. (2019). Real-time identification of blood regions for hemostasis support in laparoscopic surgery. Signal Image Video Process..

[27] Jo K., Choi B., Choi S., Moon Y., Choi J. Automatic detection of hemorrhage and surgical instrument in laparoscopic surgery image. Proceedings of the 2016 38th Annual International Conference of the IEEE Engineering in Medicine and Biology Society (EMBC).

[28] Kugener G., Zhu Y., Pangal D.J., Sinha A., Markarian N., Roshannai A., Chan J., Anandkumar A., Hung A.J., Wrobel B.B. (1906). Deep neural networks can accurately detect blood loss and hemorrhage control task success from intraoperative video. Neurosurgery.

[29] Pangal D.J., Kugener G., Zhu Y., Sinha A., Unadkat V., Cote D.J., Strickland B., Rutkowski M., Hung A., Anandkumar A. (2022). Expert surgeons and deep learning models can predict the outcome of surgical hemorrhage from 1 min of video. Sci. Rep..

[30] Lecoanet A., Vidal-Trecan G., Prate F., Quaranta J.-F., Sellier E., Guyomard A., Seigneurin A., François P. (2016). Assessment of the contribution of morbidity and mortality conferences to quality and safety improvement: A survey of participants’ perceptions. BMC Health Serv. Res..

[31] Jin A., Yeung S., Jopling J., Krause J., Azagury D., Milstein A., Fei-Fei L. Tool detection and operative skill assessment in surgical videos using region-based convolutional neural networks. Proceedings of the 2018 IEEE Winter Conference on Applications of Computer Vision (WACV).

[32] Yamazaki Y., Kanaji S., Matsuda T., Oshikiri T., Nakamura T., Suzuki S., Hiasa Y., Otake Y., Sato Y., Kakeji Y. (2020). Automated surgical instrument detection from laparoscopic gastrectomy video images using an open source convolutional neural network platform. J. Am. Coll. Surg..

[33] Lee D., Yu H.W., Kwon H., Kong H.-J., Lee K.E., Kim H.C. (2020). Evaluation of surgical skills during robotic surgery by deep learning-based multiple surgical instrument tracking in training and actual operations. J. Clin. Med..

[34] Cacciamani G.E., Anvar A., Chen A., Gill I., Hung A.J. (2021). How the use of the artificial intelligence could improve surgical skills in urology: State of the art and future perspectives. Curr. Opin. Urol..

[35] Checcucci E., Autorino R., Cacciamani G.E., Amparore D., De Cillis S., Piana A., Piazzolla P., Vezzetti E., Fiori C., Veneziano D. (2020). Artificial intelligence and neural networks in urology: Current clinical applications. Minerva Urol. Nefrol..

[36] Chen A.B., Haque T., Roberts S., Rambhatla S., Cacciamani G., Dasgupta P., Hung A.J. (2022). Artificial Intelligence Applications in Urology: Reporting Standards to Achieve Fluency for Urologists. Urol. Clin. N. Am..

[37] Gómez Rivas J., Toribio Vázquez C., Ballesteros Ruiz C., Taratkin M., Marenco J.L., Cacciamani G.E., Checcucci E., Okhunov Z., Enikeev D., Esperto F. (2021). Artificial intelligence and simulation in urology. Actas Urol. Esp. (Engl. Ed.).

[38] Hung A.J., Chen A.B., Cacciamani G.E., Gill I.S. (2021). Artificial Intelligence Will (MAY) Make Doctors Expendable (IN GOOD WAYS). Pro. Eur. Urol. Focus..

[39] Anh N.X., Nataraja R.M., Chauhan S. (2020). Towards near real-time assessment of surgical skills: A comparison of feature extraction techniques. Comput. Methods Programs Biomed..

[40] Zia A., Sharma Y., Bettadapura V., Sarin E.L., Essa I. (2018). Video and accelerometer-based motion analysis for automated surgical skills assessment. Int. J. Comput. Assist. Radiol. Surg..

[41] Azari D.P., Frasier L.L., Quamme S.R.P., Greenberg C.C., Pugh C., Greenberg J.A., Radwin R.G. (2019). Modeling surgical technical skill using expert assessment for automated computer rating. Ann. Surg..

[42] Hung A.J., Chen J., Jarc A., Hatcher D., Djaladat H., Gill I.S. (2018). Development and validation of objective performance metrics for robot-assisted radical prostatectomy: A pilot study. J. Urol..

[43] Levin M., McKechnie T., Khalid S., Grantcharov T.P., Goldenberg M. (2019). Automated methods of technical skill assessment in surgery: A systematic review. J. Surg. Educ..

[44] Aminsharifi A., Irani D., Pooyesh S., Parvin H., Dehghani S., Yousofi K., Fazel E., Zibaie F. (2017). Artificial neural network system to predict the postoperative outcome of percutaneous nephrolithotomy. J. Endourol..

[45] Murff H.J., FitzHenry F., Matheny M.E., Gentry N., Kotter K.L., Crimin K., Dittus R.S., Rosen A.K., Elkin P.L., Brown S.H. (2011). Automated identification of postoperative complications within an electronic medical record using natural language processing. JAMA.

[46] Bruce J., Russell E., Mollison J., Krukowski Z. (2002). The measurement and monitoring of surgical adverse events. Clin. Gov..

[47] Hamilton E.C., Pham D.H., Minzenmayer A.N., Austin M.T., Lally K.P., Tsao K., Kawaguchi A.L. (2018). Are we missing the near misses in the OR?—Underreporting of safety incidents in pediatric surgery. J. Surg. Res..

[48] Seiden S.C., Barach P. (2006). Wrong-side/wrong-site, wrong-procedure, and wrong-patient adverse events: Are they preventable?. Arch. Surg..

[49] Elwy A.R., Itani K.M., Bokhour B.G., Mueller N.M., Glickman M.E., Zhao S., Rosen A.K., Lynge D., Perkal M., Brotschi E.A. (2016). Surgeons’ disclosures of clinical adverse events. JAMA Surg..

[50] Alsubaie H., Goldenberg M., Grantcharov T. (2019). Quantifying recall bias in surgical safety: A need for a modern approach to morbidity and mortality reviews. Can. J. Surg..

[51] Feldman L., Barkun J., Barkun A., Sampalis J., Rosenberg L. (1997). Measuring postoperative complications in general surgery patients using an outcomes-based strategy: Comparison with complications presented at morbidity and mortality rounds. Surgery.

[52] Hutter M.M., Rowell K.S., Devaney L.A., Sokal S.M., Warshaw A.L., Abbott W.M., Hodin R.A. (2006). Identification of surgical complications and deaths: An assessment of the traditional surgical morbidity and mortality conference compared with the American College of Surgeons-National Surgical Quality Improvement Program. J. Am. Coll. Surg..

