# Supplementary material for: Automated Capture of Intraoperative Adverse Events Using Artificial Intelligence: A Systematic Review and Meta-Analysis
Source: J Clin Med. 2023 Feb 20;12(4):1687. doi: 10.3390/jcm12041687 (PMC9963108; doi:10.3390/jcm12041687)
Supplement: Supplementary file 1 [file jcm-12-01687-s001.zip › Figure S3.pdf]

Figure S3: Flowcharts of Methodologies for Automatically Detecting Intraoperative Adverse Events [17–29].

Chen et al (2021)	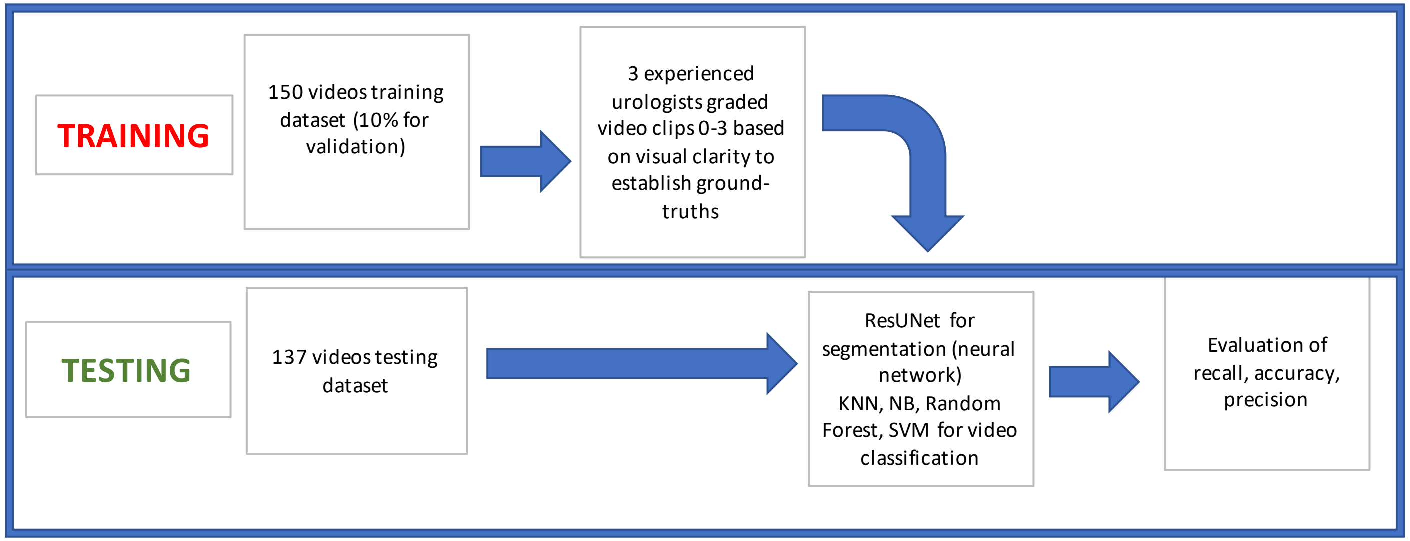

Morita et al (2020)	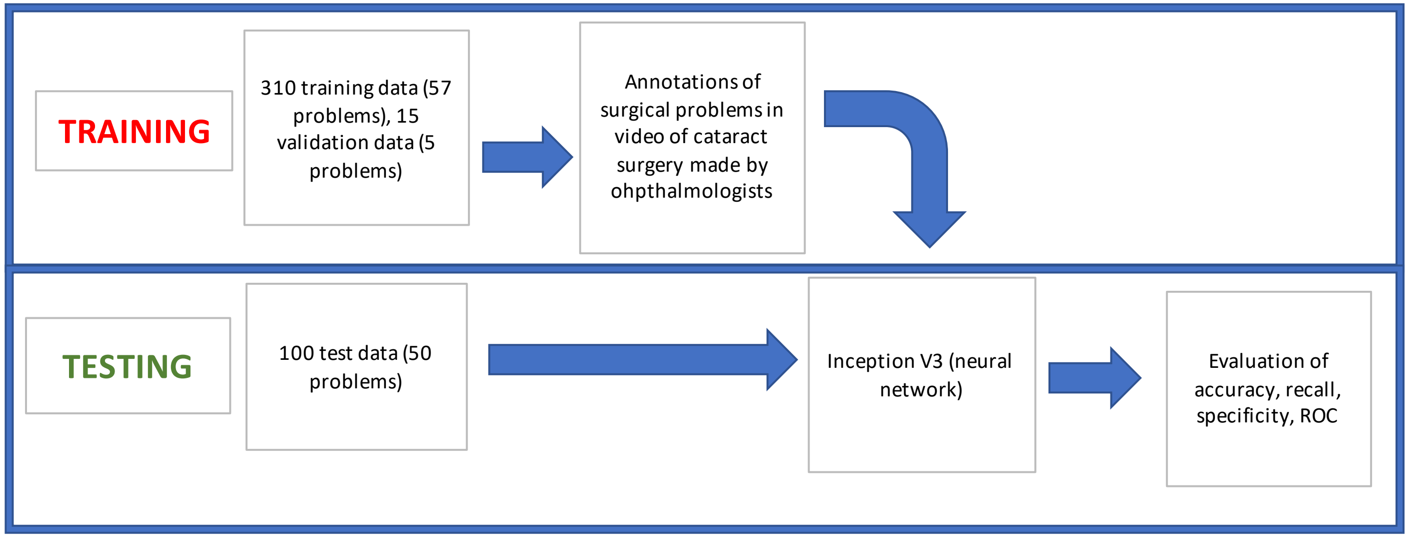

Park et al (2020)	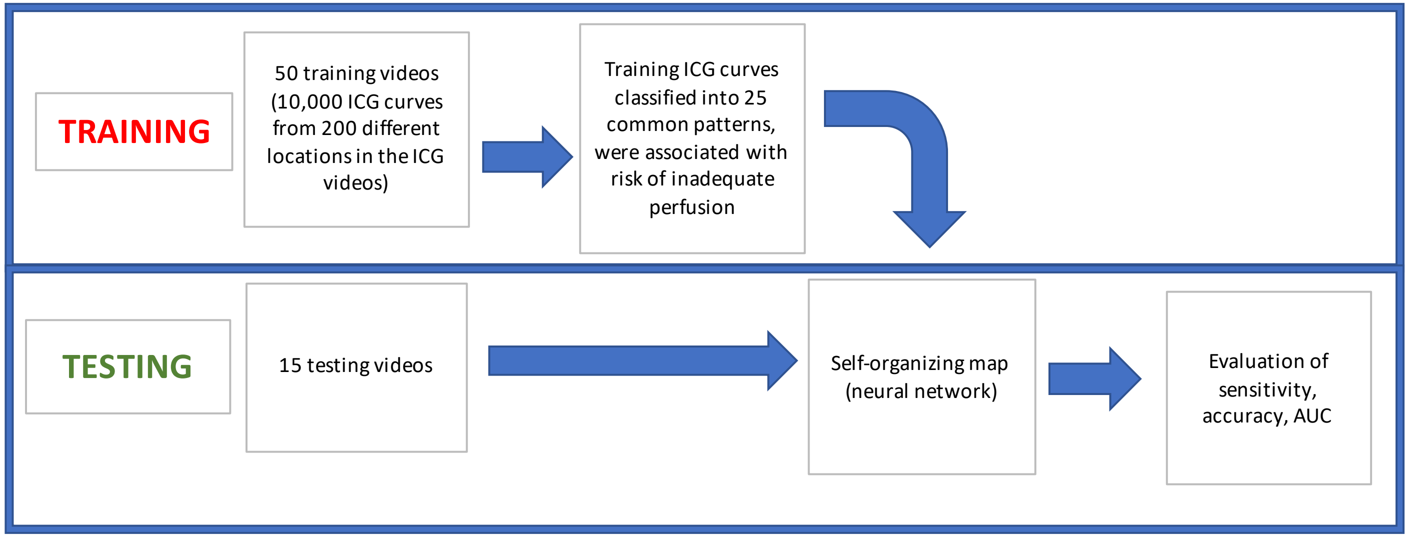

Su et al (2022)

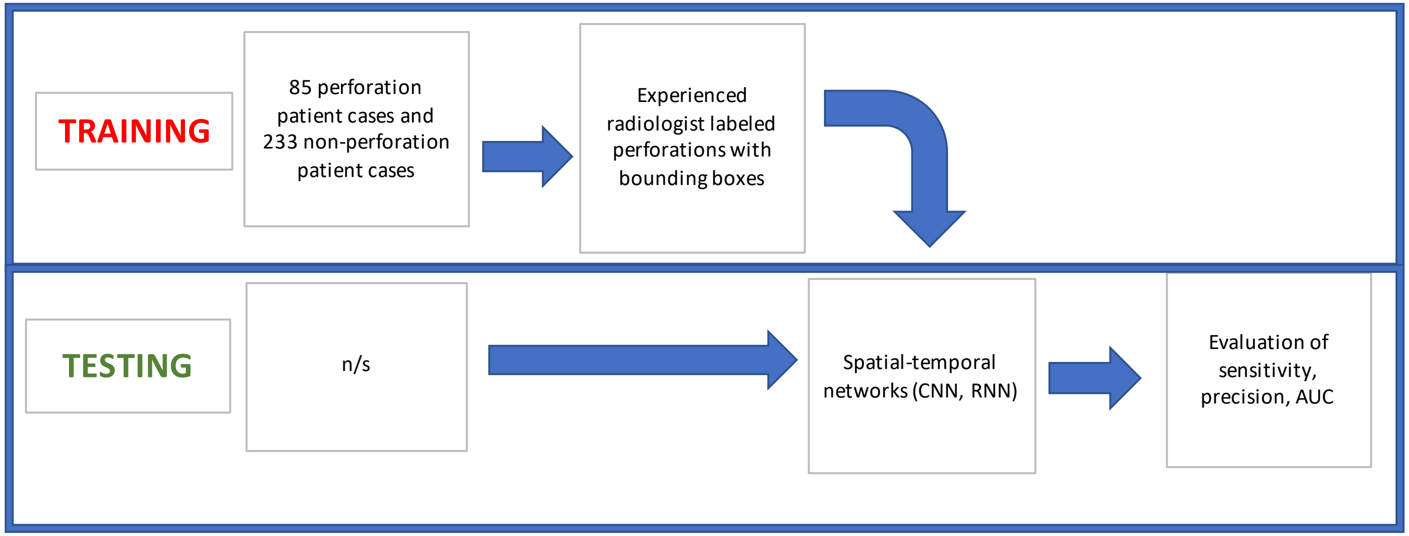
TRAINING
85 perforation patient cases and 233 non-perforation patient cases
Experienced radiologist labeled perforations with bounding boxes
TESTING
n/s
Spatial-temporal networks (CNN, RNN)
Evaluation of sensitivity, precision, AUC

Zhang et al (2019)	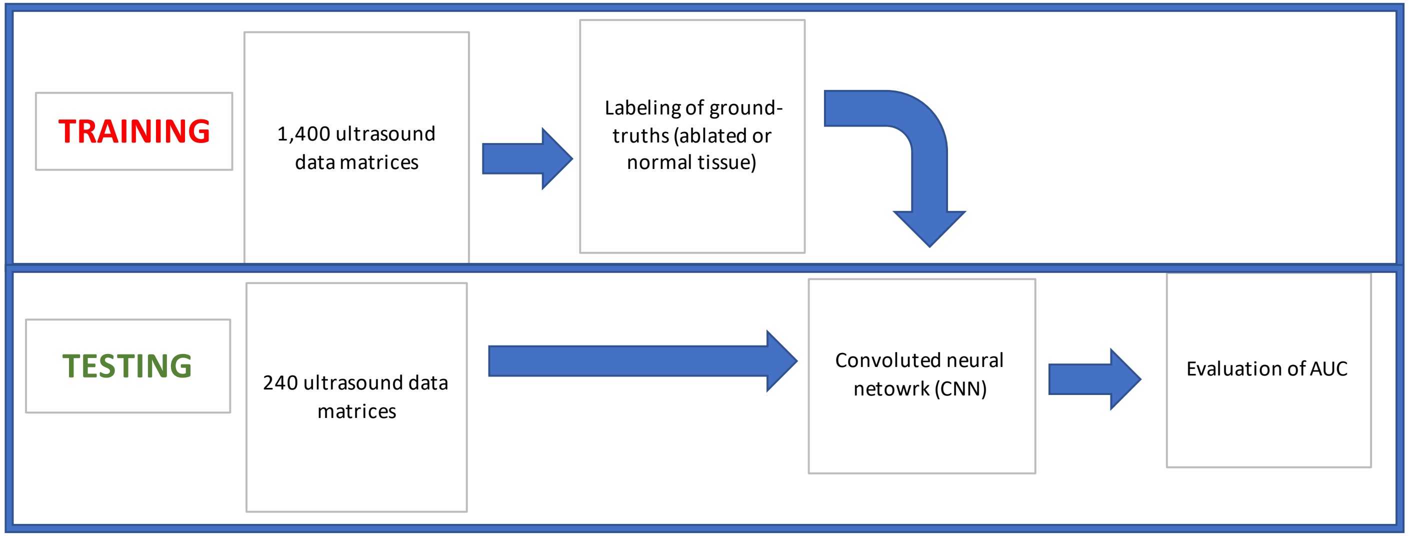

Zha et al (2020)	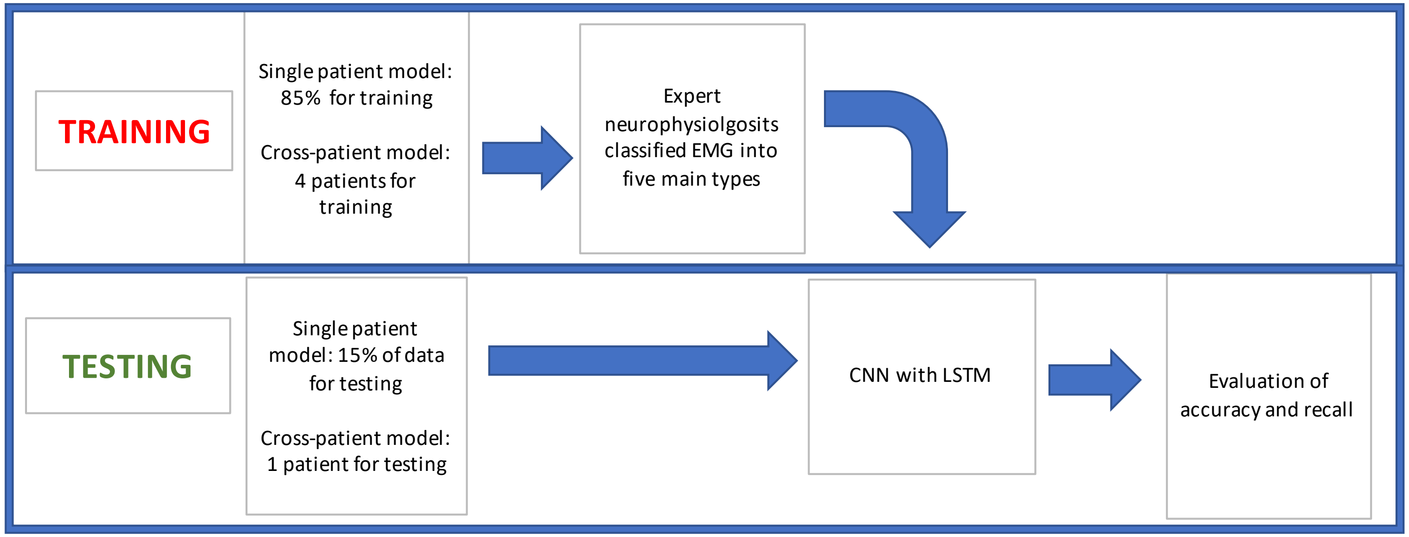

Garcia-Martinez et al (2017)	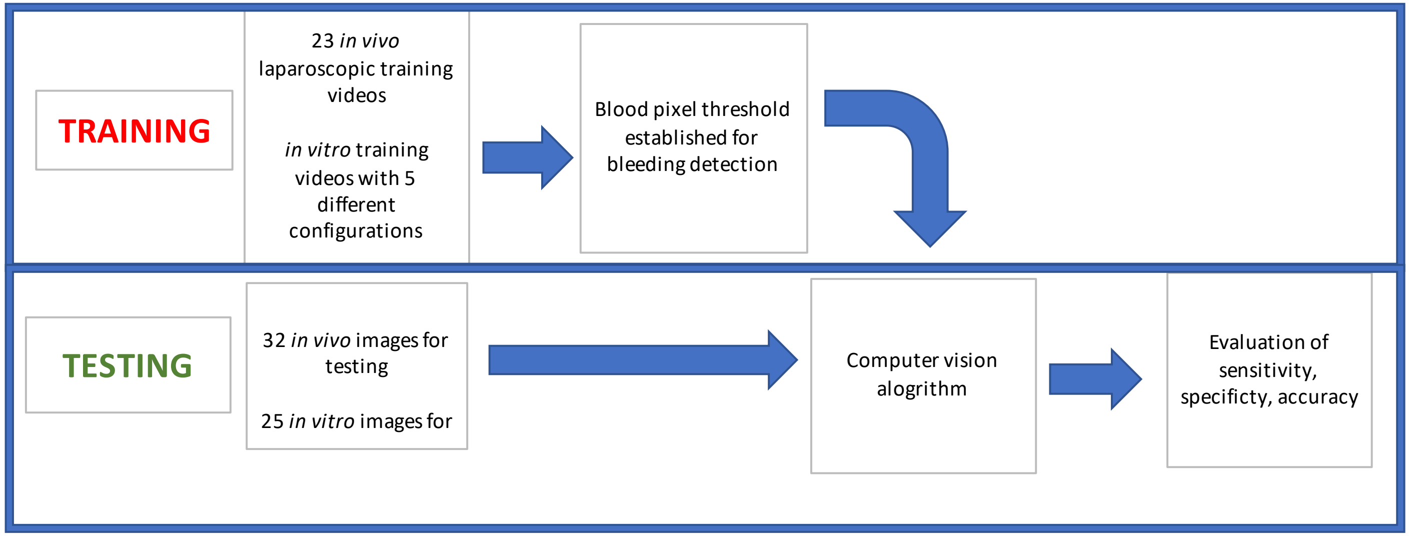

Wei et al (2021)	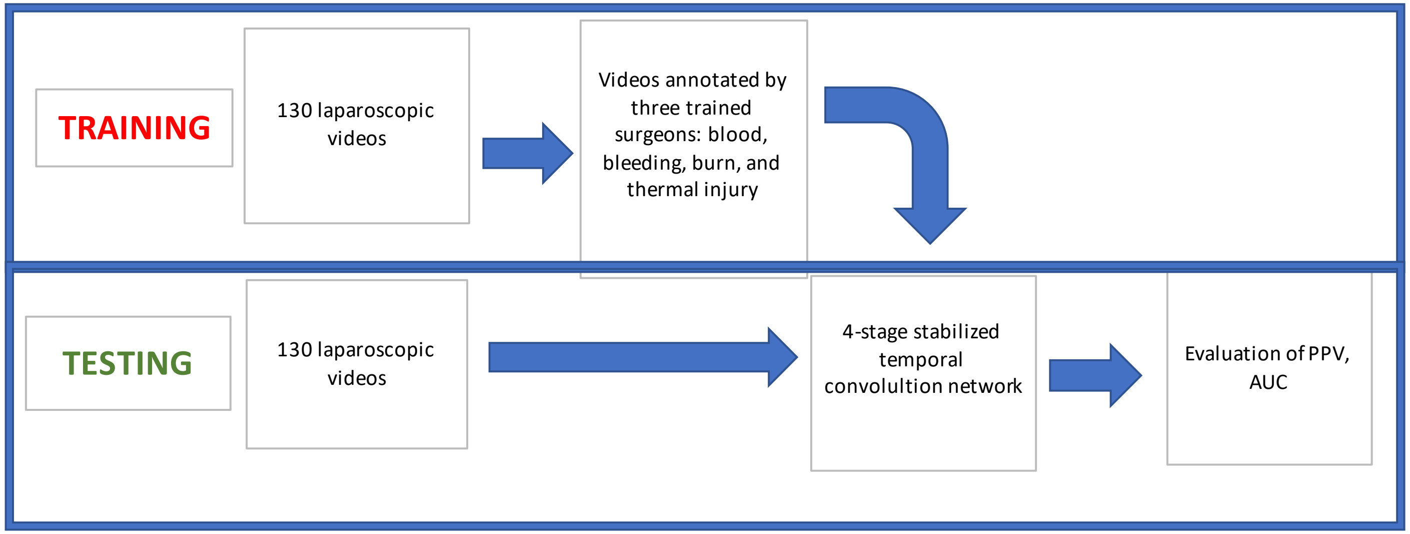

Hua et al (2022)

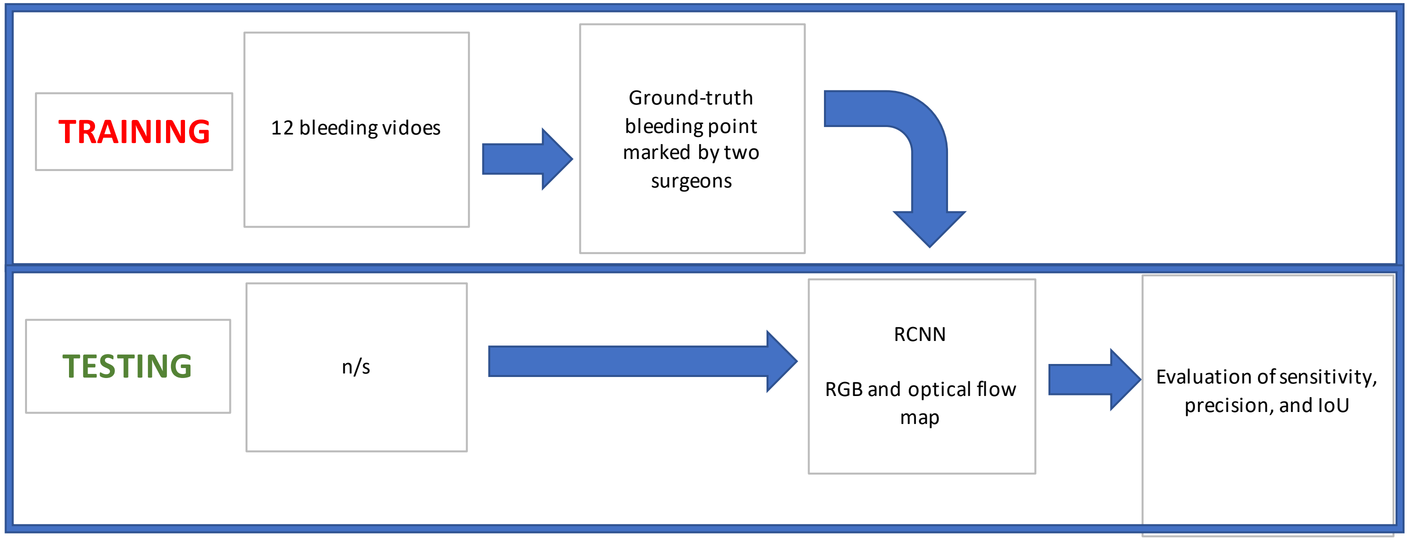

Okamoto et al (2019)	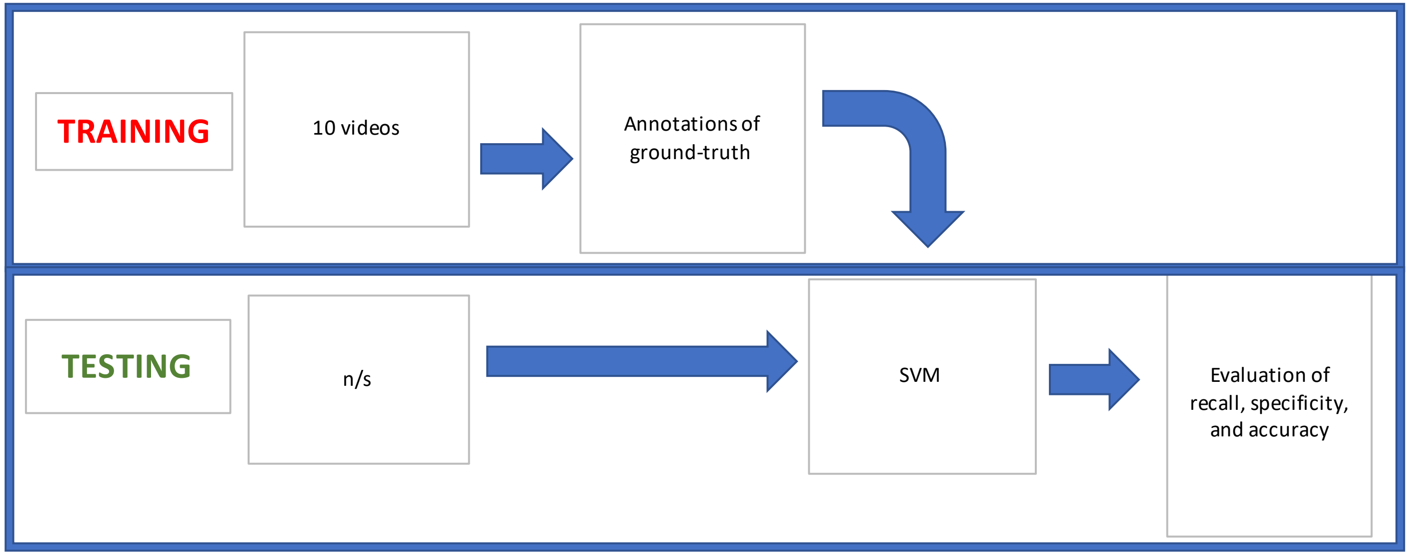

Jo et al (2016)	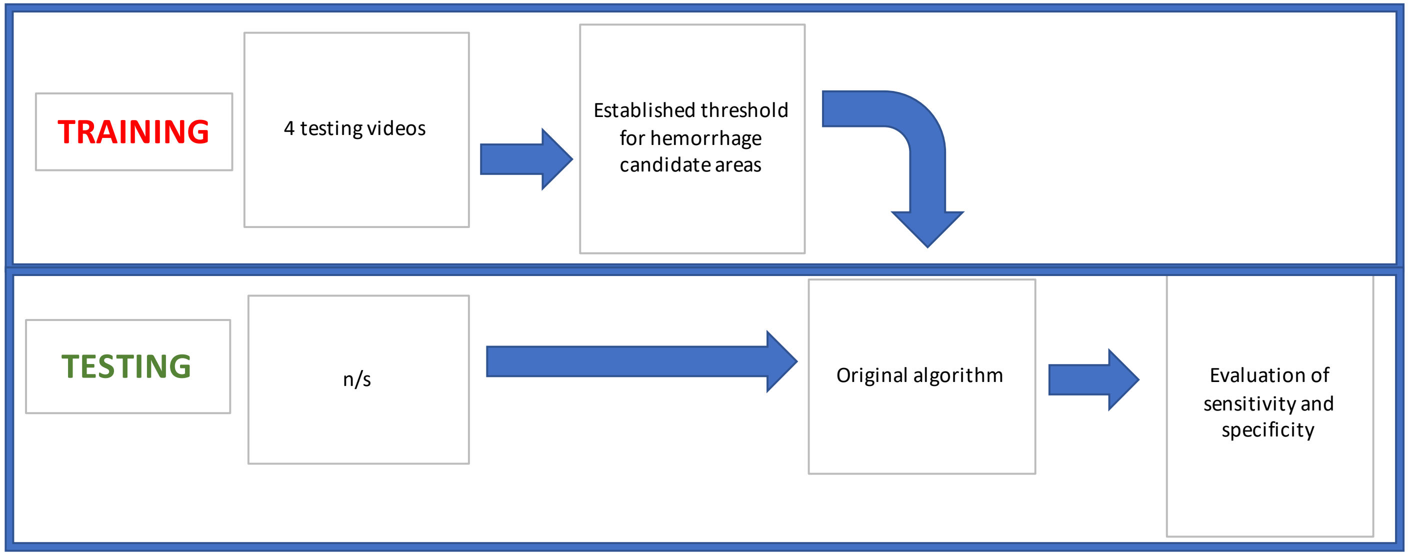

TRAINING
4 testing videos
Established threshold for hemorrhage candidate areas
TESTING
n/s
Original algorithm
Evaluation of sensitivity and specificity

Kugener et al(2022)	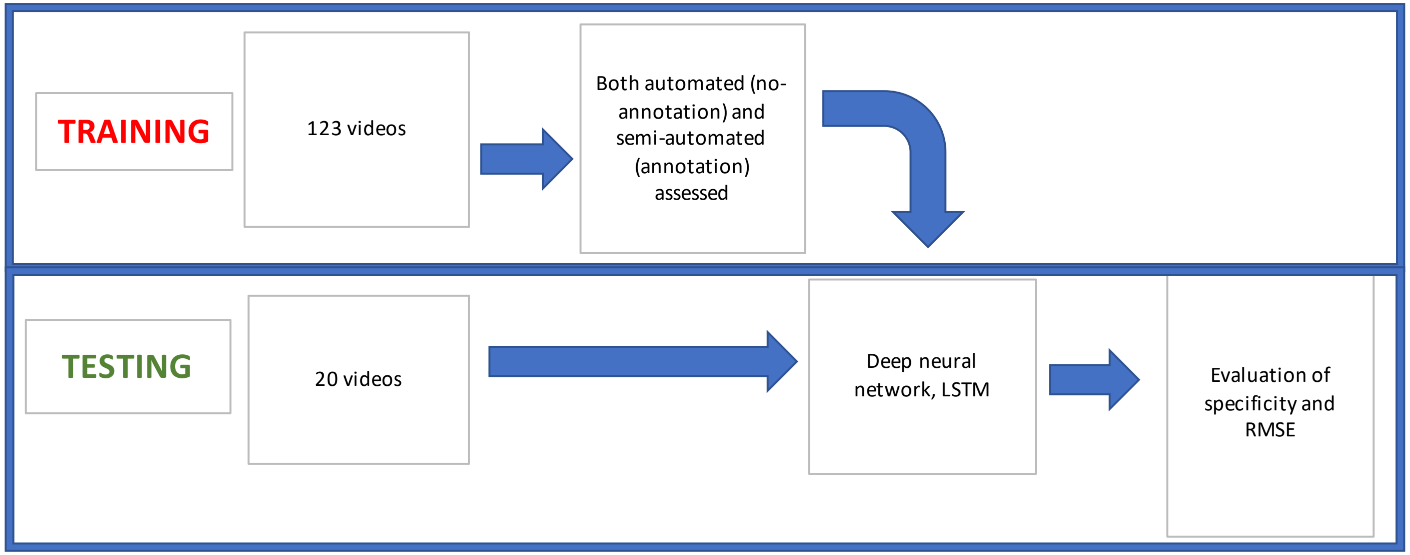

Pangal et al (2022)	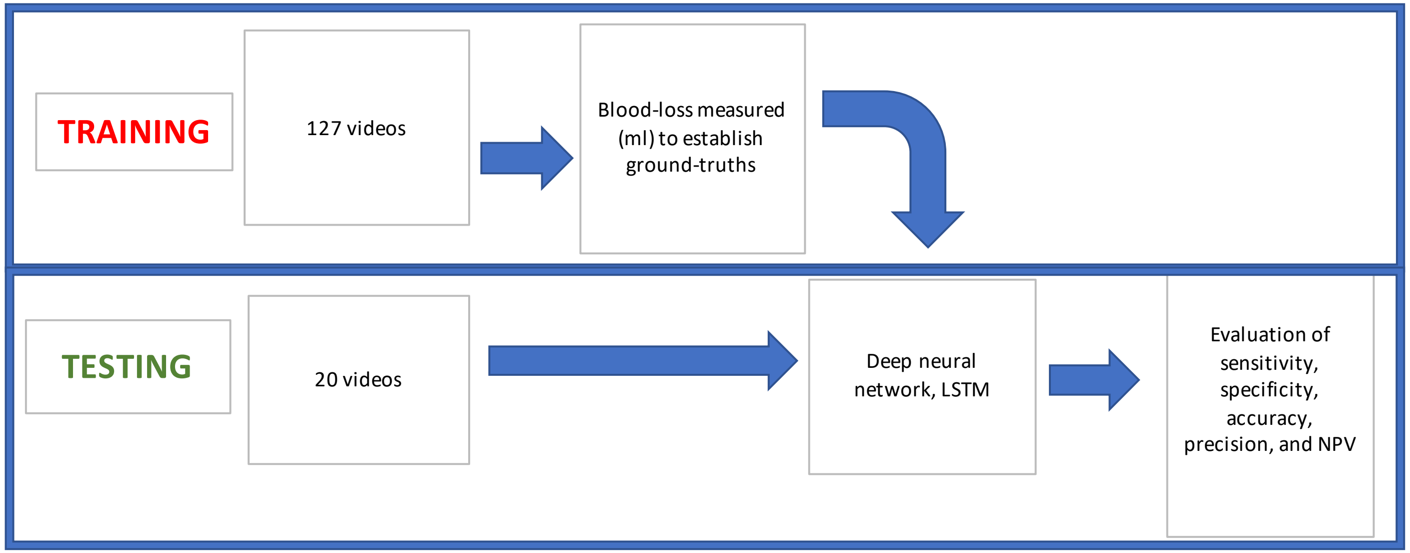
