# Supplementary material for: Automated Capture of Intraoperative Adverse Events Using Artificial Intelligence: A Systematic Review and Meta-Analysis
Source: J Clin Med. 2023 Feb 20;12(4):1687. doi: 10.3390/jcm12041687 (PMC9963108; doi:10.3390/jcm12041687)
Supplement: Supplementary file 1 [file jcm-12-01687-s001.zip › Figures S1 and S2.pdf]

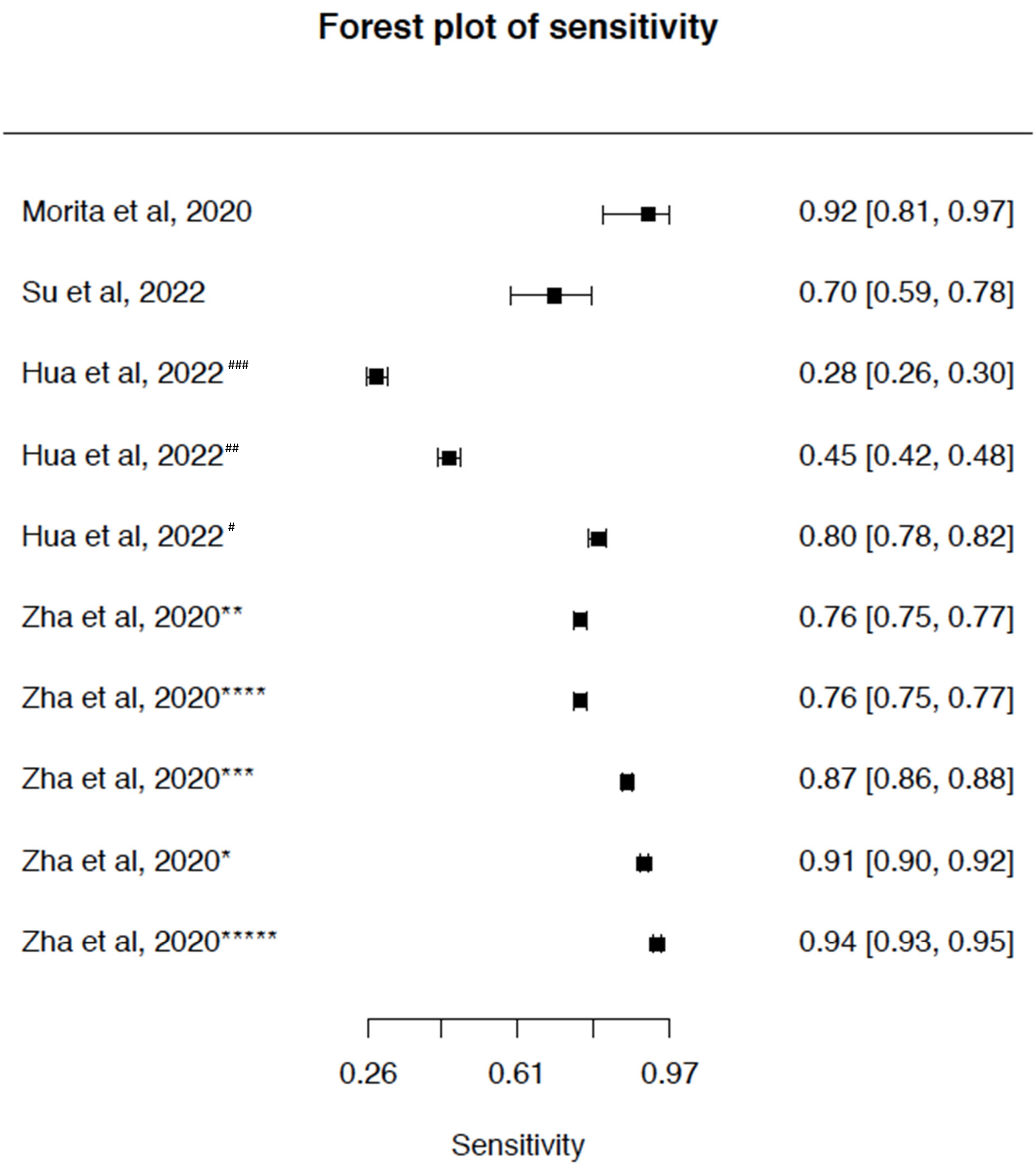

Figure S1. Forest plot of sensitivity. # Hua RGB + optical flow; ## Hua RGB; ### Hua optical flow; * Zha CNN-LSTM single patient; **Zha GNB single patient; ***Zha CNN single patient; ****Zha RNN single patient; *****Zha CNN-LSTM cross patient [18,20,22,25].

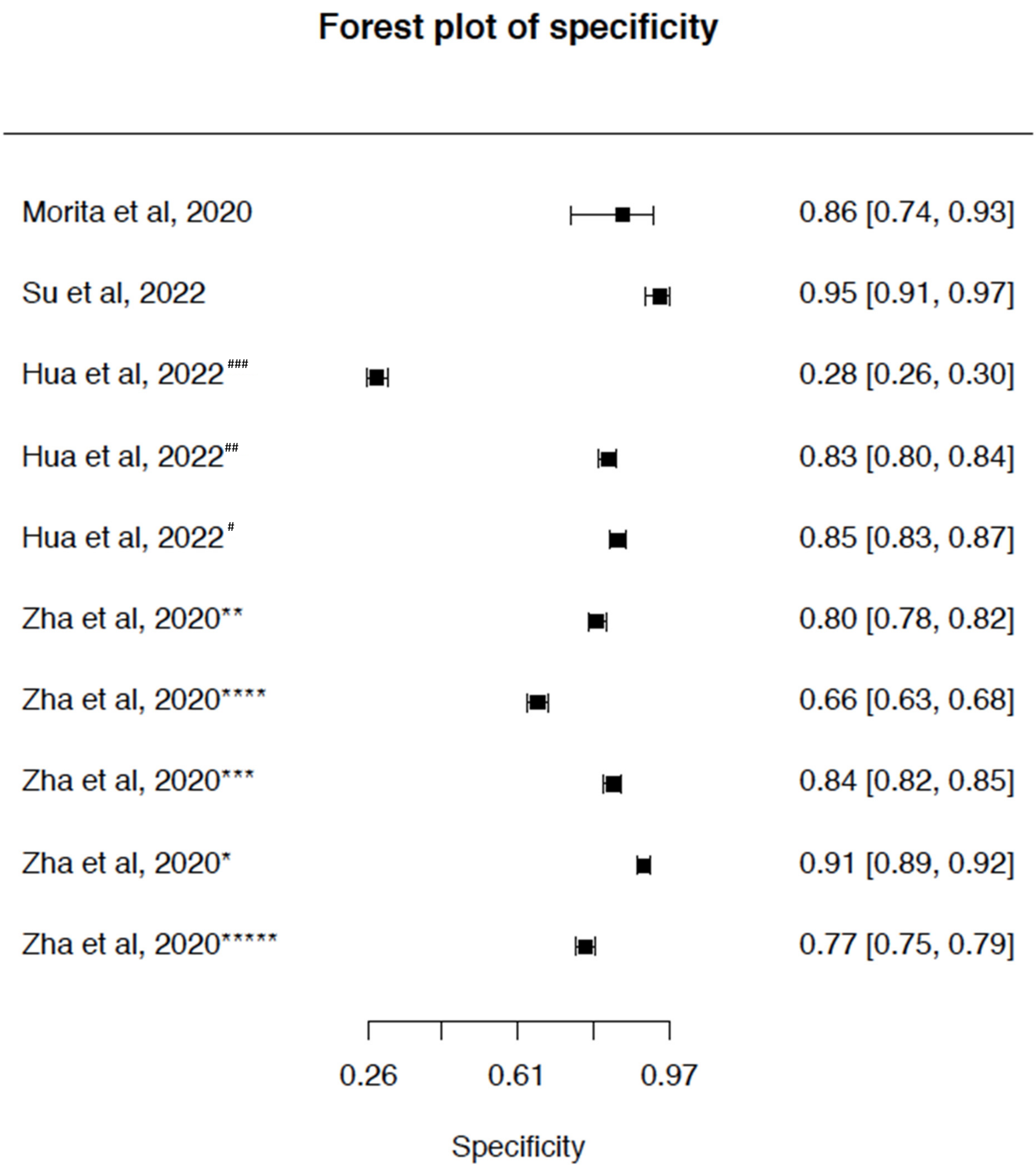

Figure S2. Forest plot of specificity. # Hua RGB + optical flow; ## Hua RGB; ### Hua optical flow; * Zha CNN-LSTM single patient; **Zha GNB single patient; ***Zha CNN single patient; ****Zha RNN single patient; *****Zha CNN-LSTM cross patient [18,20,22,25].
