# Supplementary material for: Automated Capture of Intraoperative Adverse Events Using Artificial Intelligence: A Systematic Review and Meta-Analysis
Source: J Clin Med. 2023 Feb 20;12(4):1687. doi: 10.3390/jcm12041687 (PMC9963108; doi:10.3390/jcm12041687)
Supplement: Supplementary file 1 [file jcm-12-01687-s001.zip › Table S1.pdf]

Search Terms by Database	
Database	Search
**PubMed**	(predict* OR identif* OR recog*) AND ((("Artificial Intelligence"[Mesh] OR "Big Data"[Mesh] OR "data science∗"[tiab] OR "data analytic∗"[tiab] OR "data-driven science∗"[tiab] OR "artificial intelligence"[tiab] OR "machine learning"[tiab] OR "big data"[tiab] OR "computational intelligence"[tiab] OR "deep learning"[tiab] OR "computer calculated"[tiab]) AND ("Surgical Procedures, Operative"[Mesh] OR "surgery" [Subheading] OR "General Surgery"[Mesh] OR "surger∗"[tiab] OR "surgical∗"[tiab] OR "perioperat∗"[tiab] OR "perioperati∗"[tiab])) AND (((((adverse event) OR (complication)) OR (patient safety incident)) OR (incident))) AND (2013:2022[pdat]))
**Scopus**	TITLE-ABS-KEY (((predict* OR identif* OR recog*) AND ((("Artificial Intelligence" OR "Big Data" OR "data science∗" OR "data analytic∗" OR "data-driven science∗" OR "artificial intelligence" OR "machine learning" OR "big data" OR "computational intelligence" OR "deep learning" OR "computer calculated") AND ("Surgical Procedures, Operative" OR "surgery" OR "General Surgery" OR "surger∗" [OR "surgical∗" OR "perioperat∗" OR "perioperati∗")) AND (((((adverse AND event) OR (complication)) OR (patient AND safety AND incident)) OR (incident))))))
**Web of Science**	AB=((predict* OR identif* OR recog*) AND ("Artificial Intelligence" OR "Big Data" OR "data science∗" OR "data analytic∗" OR "data-driven science∗" OR "artificial intelligence" OR "machine learning" OR "big data" OR "computational intelligence" OR "deep learning" OR "computer calculated") AND ("Surgical Procedures, Operative" OR "surgery" OR "General Surgery" OR "surger∗" OR "surgical∗" OR "perioperat∗" OR "perioperati∗") AND ("adverse event" OR complication OR "patient safety incident" OR incident))
**IEEE Xplore**	(((Artificial Intelligence OR Big Data OR data science OR data analytic OR data-driven science OR artificial intelligence OR machine learning OR big data OR computational intelligence OR deep learning OR computer calculated) AND (Surgical Procedures OR surgery OR General Surgery OR surger_ OR surgical_ OR perioperat_ OR perioperati_) AND (adverse event OR complication OR patient safety incident OR incident)))
