# Supplementary material for: Automated Capture of Intraoperative Adverse Events Using Artificial Intelligence: A Systematic Review and Meta-Analysis
Source: J Clin Med. 2023 Feb 20;12(4):1687. doi: 10.3390/jcm12041687 (PMC9963108; doi:10.3390/jcm12041687)
Supplement: Supplementary file 1 [file jcm-12-01687-s001.zip › Table S2.pdf]

Study	Algorithm or statistic model	Description	Sensitivity (recall)	Specificy	Accuracy	PPV (precision)	NPV	AUC-ROC	IoU	Dice Coefficent	Correlation coefficient	RMSE	Time consumption
Chen	KNN	Validation			0.55								
	Naïve Bayes	Validation			0.64								
	Random Forest	Validation			0.73								
	SVM	Validation			0.64								
	KNN	Testing (revised)	0.89		0.9	0.85							
	Naïve Bayes	Testing (revised)	0.9		0.89	0.85							
	Random Forest	Testing (revised)	0.92		0.9	0.86							
	SVM	Testing (revised)	0.91		0.91	0.87							
	ResUNet (segmentation)								0.51	0.69			
	KNN (w/ segmentation)	Correlation (testing and ground-truth)									0.59		
	Naïve Bayes (w/ segmentation)	Correlation (testing and ground-truth)									0.62		
	Random Forest (w/ segmentation)	Correlation (testing and ground-truth)									0.62		
	SVM (w/ segmentation)	Correlation (testing and ground-truth)									0.6		
	KNN (w/out segmentation)	Correlation (testing and ground-truth)									0.58		
	Naïve Bayes (w/out segmentation)	Correlation (testing and ground-truth)									0.6		
	Random Forest (w/out segmentation)	Correlation (testing and ground-truth)									0.62		
	SVM (w/out segmentation)	Correlation (testing and ground-truth)									0.6		
Morita	Inception V3 (CNN)	This study	0.91	0.86	0.9			0.97					
Park	Self-organizing map (artificial neural network)	This study	1		0.87	0.6		0.84					
	TR	Conventional parameter	0.67		0.87	0.67		0.73					
	T1/2max	Conventional parameter	0.67		0.67	0.33		0.75					
	RS	Conventional parameter	1		0.8	0.5		0.68					
Su	Spatial-temporal networks (CNN, RNN)	10-fold cross validation	0.7			0.83		0.93					
Zhang	SI-CNN	This study						0.87					
	SIm-CNN	This study						0.89					
	B-mode	Other metric						0.69					
Zha	CNN-LSTM single patient	This study	0.91		0.91								
	GNB single patient	Other metric	0.76		0.75								
	CNN single patient	Other metric	0.87		0.86								
	RNN single patient	Other metric	0.76		0.73								
	CNN-LSTM cross patient	Training	0.99		0.997								
	CNN-LSTM cross patient	Testing	0.94		0.89								
Garcia-Martinez	Computer vision algorithm, in vitro	This study	0.87	0.96	0.95								
	Computer vision algorithm, in vivo	This study	0.72	0.93	0.88								
	Tonmoy Ghosh, in vitro	Other metric	0.96	0.92	0.92								
	Tonmoy Ghosh, in vivo	Other metric	0.91	0.49	0.6								
	Yixuan Yuan, in vitro	Other metric	0.996	0.83	0.84								
	Yixuan Yuan, in vivo	Other metric	0.93	0.5	0.61								
Wei	Thermal injury detection	This study				0.63		0.95					
	Bleeding detection	This study				0.34		0.82					
Hua	RGB + optical flow		0.8			0.84			0.7				
	RGB		0.45			0.72							
	Optical flow		0.28			0.28							
Okamoto	Same model laparoscope		0.95-0.99	0.91-0.995	0.91-0.99								
	Different model laparoscope		0.93-1	0.86-1	0.86-1								
Jo	Hemorrhage detection		.45-1	0.33-1									0.1-0.6 sec
Kugener	Deep learning network	This study		0.85								358	
		Control										489	
		Control										431	
Pangal	SOCALNet		1	0.66	0.85	0.79	1						

PPV positive predictive value, NPV negative predictive value, AUC-ROC aread under curve-receiver operator characteristics, RMSE root mean squre error
